# The Effect of Magnesium Concentration on Myogenic Cardiac Function: Larval *Drosophila*

**DOI:** 10.17912/micropub.biology.001400

**Published:** 2024-12-20

**Authors:** Joy Bidros, Kaitlyn Brock, Jaycie Gard, Robin Cooper

**Affiliations:** 1 Biology, University of Kentucky, Lexington, Kentucky, United States

## Abstract

The heart of larval *Drosophila* serves as a model preparation in addressing cardiac function, as known genetic mutations can be mimicked to examine therapies. Pharmacological agents and function of proteins, like TRPA1, which affect ionic transport and ion concentrations can be investigated for their action on cardiac function in this model. To maintain *in-situ* function, the larval heart tube needs to remain viable; thus, a physiological saline is required. It was found that a reduced Mg ^2+^ level from the standard saline provides a more stable heartbeat, even in stressful conditions such as heat and increased expression levels of TRPA1 proteins.

**Figure 1. The effect of Mg 2+ on the early 3 rd instar heart rate (HR) in varied conditions:**
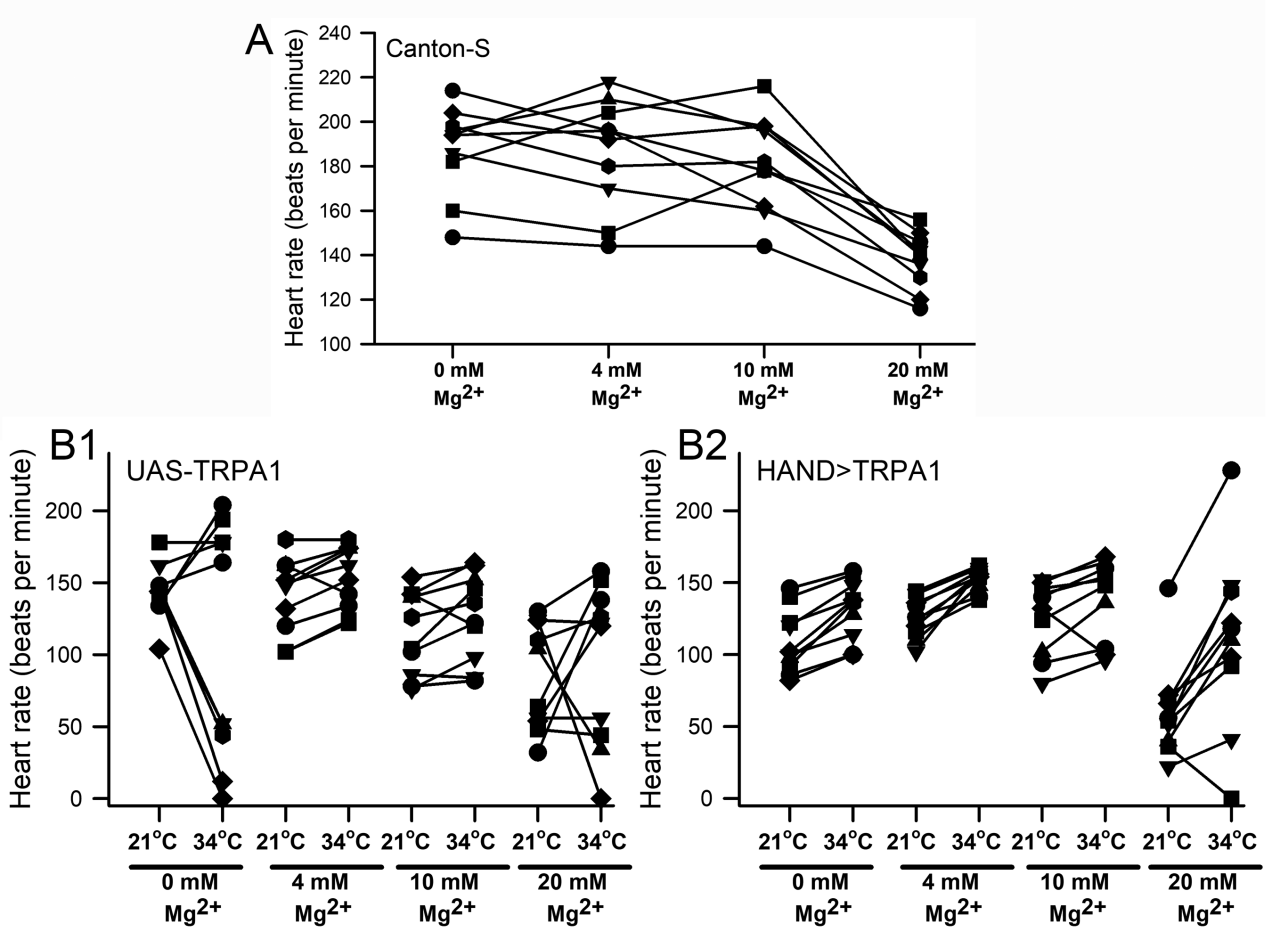
(A) The HR of individual preparations during exposure to various concentrations of Mg ^2+^ . (B1) Parental and (B2) a strain with over expression of TRPA1 receptors specifically in the heart with exposure to varied concentrations of Mg ^2+^ along with changes in temperature (from 21 to 34 ^o^ C).

## Description

The larval heart tube of *Drosophila* serves as a model preparation in addressing the myogenic properties of cardiac function. Studies on the development of the heart from an embryo to an adult have also provided valuable insights in developmental biology. Given the heart in the early larvae stages is not innervated but becomes innervated in the late third instar, direct assessment can be performed to examine how cardiovascular modulators affect the rhythmicity. This aids in the assessment of pharmacological agents. The pioneering research of Sidney Ringer in developing a saline to maintain the physiological functions of the frog heart led to many advances in medicine and health. Given that the *Drosophila* model offers a genetically amendable model to address many disease states affecting humans, development of a physiological saline which best maintains the cardiac function is imperative for continuing future research using this preparation.

Development of a physiological saline for maintaining synaptic transmission at the neuromuscular junction (NMJ) of the body wall muscles in larval and adult *Drosophila* has taken years. The saline used today for physiological recordings at the NMJ has undergone many iterations in adjusting buffers and ionic concentrations to maintain synaptic function for periods of 30 minutes or more. The ionic composition of the larval hemolymph was established with ion sensitive electrodes which led the way to prolonged maintenance of synaptic transmission and membrane potential of the larval body wall muscles (Stewart et al., 1994) . However, these earlier salines did not allow the larval heart to maintain beating in-situ for more than several minutes. Thus, it is difficult to obtain physiological measures in-situ. This is one reason pupa and intact larvae are used to examine genetic alteration in cardiac performance. Pupa offer an advantage that they do not crawl; however, intact larvae must be restrained to clearly observe the heart tube for periods of time. The pupal stage is a time of transition in the heart’s morphology and tissue development as well as neural innervation. It is also a time of changes in ionic, peptide, and cardiomodulating content of the hemolymph. Restraining larvae can also be stressful (Cooper et al., 2009) . The intact organism does not allow pharmacological agents to be applied with ease, and the physical stress would likely have an effect in the release of modulators within the hemolymph. It has been shown that with ontogenetically stimulating serotoninergic, dopaminergic or cholinergic neurons in restrained intact larvae the rate of the heartbeat can be affected (Malloy et al., 2017) .

Dissected early third instar larval preparations allow a defined bathing environment free from hormones and peptides in the hemolymph, without neural regulation, and a known composition of saline to be used, all of which reduces experimental variability. The HL3 salines previously developed do not allow the heartbeat to be maintained for longer than several minutes (de Castro et al., 2014, 2019) . Since the larval heartbeat is dependent on the extracellular Ca ^2+^ concentration, and that Mg ^2+^ is well established to block Ca ^2+^ channels, it is possible that also reducing the Mg ^2+^ concentration in the saline used for the larval heart might improve function. It is also important to be able to measure function in varied conditions to examine potential treatments in pathologies as well as potential pharmacological agents. As a start in this endeavor, we examined the effects of a modified saline in larvae which are over expressing the TRPA1 receptor, specifically in the heart. The action of the TRPA1 receptors in the cardiovascular system is still being investigated, but there have been implications in the pathogenesis of cardiovascular disorders (Pazienza et al., 2014) . The *Drosophila* TRPA1 channels detect heat (Wang et al., 2013) and can likely detect stretch of the membrane (Bodkin et al., 2014; Andrei et al., 2017) . In mammals, TRPA is expressed in tissues associated with the heart (vascular smooth muscle, endothelial cells, myocytes) and is altered in expression with cardiac injury and ischemia. Enhancing expression in pathological conditions may be a compensatory mechanism to increase contractility during stretch (Andrei et al., 2017) .

Understanding the effect of varying concentration of Mg ^2+^ in salines used for the larval *Drosophila* heart under different conditions and modifications will allow improvements in future physiological investigations using this model preparation.

The concentration of Mg ^2+^ in the saline had an impact on the basal heart rate and on the degree of change in the rate depending on the conditions. The 0, 4, and 10 mM of Mg ^2+^ showed a higher heart rate as compared to 20 mM at 21 ^0^ C (ANOVA; P<0.05). When the hearts were exposed to 4 or 10 mM Mg ^2+^ and given a heat stress, the rates showed an increase (Paired T-test, P<0.5) while the 0 and 20 mM Mg ^2+^ did not provide a consistent response with some preparations stopping the heart beat entirely. In addition, 5 out of 10 preparations in both conditions of 0 and 20 mM Mg ^2+^ increased in their rate while the other half decreased in their rate with the change from 21 ^0^ C to 34 ^0^ C. However, when expressing the TRPA1 protein in the heart the 0, 4, 10 and 20 mM of Mg ^2+^ all showed a significant increase in the heart rate for the 21 ^0^ C to 34 ^0^ C temperature change (Paired T-test, P<0.05). Only one preparation at 10 mM and one for the 20 mM Mg ^2+^ presented with a decrease in the rate. The change was more pronounced for the 20 mM Mg ^2+^ due to starting at a lower rate initially at 21 ^0^ C. Although, the maximum rates of the preparations at 34 ^0^ C for 20 mM Mg ^2+^ were still significantly lower than for the maximum rates for the preparations exposed to 4 mM Mg ^2+^ (ANOVA, P<0.05).

As the continual development of salines to be used for physiological measures of skeletal and cardiac muscles in many model preparations (crustacean, amphibian, mammals) progresses, so shall the saline for maintaining cardiac function in the *Drosophila* model. It was noted that a saline which mimics HL3 composition, but with reduced Mg ^2+^ , provides a prolonged maintenance in the amplitude of the excitatory junction potential (EJP) at the NMJ for larval *Drosophila* (Feng et al., 2004) . Likewise, a lowered Mg ^2+^ from 20 mM to 4 mM appears to be preferred for physiological indexing of cardiac performance in the larvae heart. Future studies are underway in our group to measure transmembrane potentials in the cardiac muscle of larval *Drosophila* with saline at 4 mM Mg ^2+^ to compare to earlier reports (Desai-Shah et al., 2010) of electrical recordings made with 20 mM to address the effects on the properties of the electrical signals.

## Methods

The common "wild-type" laboratory strain of *D. melanogaster,* Canton S, and overexpression in the heart was performed by crossing female virgins of UAS-TrpA1 (BDSC stock # 26263) with males of heart-specific strains, Hand4.2-Gal4 (on II) to produce Hand4.2>TrpA1. The heart-specific line (Hand4.2-Gal4), was supplied by Dr. Anthony Cammarato. The expression of GAL4 system starts to occur during embryonic development (Brand and Perrimon, 1993) when the heart tube develops to express Hand4.2 then TRPA1 will be expressed. The GFP in HAND::GFP can be observed as early as embryonic stage 14 (Tiwari et al., 2021) .

Only early 3rd instar larvae were used (50-70 h post hatching). All larvae were maintained at room temperature 21 ^O^ C in vials partially filled with a cornmeal-agar-dextrose-yeast medium. The larval dissection technique to expose the larval heart tubes have been previously reported in video format (Cooper et al., 2009) .

A modified HL3 saline was used (NaCl 70 mM, KCl 5 mM, MgCl _2_ .6H _2_ O (either 0 mM, 4 mM, 10 mM or 20 mM), NaHCO _3_ 10 mM, Trehalose 5 mM, sucrose 115 mM, BES (N,N-bis(2-hydroxyethyl)-2-aminoethanesulfonic acid) 25 mM, and CaCl _2_ .2H _2_ O 1 mM, with pH 7.1). The chemicals were obtained from Sigma-Aldrich, St. Louis, MO, USA. The glass dissection dish was placed on top of a submerged platform in water maintained at either 34°C or on a hollowed copper plate with antifreeze perfusion from a temperature-controlled incubator at 21°C (Marguerite et al., 2021) . Each exposure time was first allowed 1 min for heat to transfer through the glass to the bathing saline. The number of heart beats were recorded first at 21°C and then at 33°C.

## Reagents

NaCl 

KCl 5

MgCl _2_ .6H _2_ O

NaHCO _3_

L-Trehalose

sucrose

N,N-bis(2-hydroxyethyl)-2-aminoethanesulfonic acid

CaCl _2_ .2H _2_ O

All from Sigma-Aldrich, St. Louis, MO, USA. 
